# The Impact of Gastroesophageal Reflux Disease on Quality of Life Among Obese Individuals in the Eastern Province of Saudi Arabia

**DOI:** 10.7759/cureus.85577

**Published:** 2025-06-08

**Authors:** May A Alharbi, Lama S Alahmadi, Faris Altom, Fahad W Raedi, Taif A Alahmadi, Shahed Chamsi Basha, Waleed Alkanderi, Afrah Almutairi, Thamraa N Jalfan, Hebah A Alaamri

**Affiliations:** 1 College of Medicine, Qassim University, Buraydah, SAU; 2 Department of Medicine, Al-Rayyan College, Madinah, SAU; 3 College of Pharmacy, Umm Al-Qura University, Mecca, SAU; 4 College of Medicine, Taibah University, Madinah, SAU; 5 College of Medicine, Al-Maarefa University, Diriyah, SAU; 6 College of Medicine, University of Jordan, Amman, JOR; 7 Department of Medicine and Surgery, King Abdulaziz University, Jeddah, SAU; 8 College of Medicine, King Khalid University, Abha, SAU; 9 College of Medicine, Imam Abdulrahman bin Faisal University, Dammam, SAU

**Keywords:** eastern province, gastroesophageal reflux disease (gerd), obesity, quality of life (qol), saudi arabia

## Abstract

Background

Gastroesophageal reflux disease (GERD) is a widespread digestive condition globally, with obesity being a significant risk factor. GERD symptoms, such as heartburn and acid reflux, are caused by abnormal relaxation of the lower esophageal sphincter (LES), allowing stomach acid to flow back into the esophagus. Chronic symptoms can negatively impact quality of life (QoL) and may lead to complications such as esophageal adenocarcinoma.

Objectives

To assess the prevalence of GERD in the Eastern Province of Saudi Arabia among obese and non-obese individuals and to examine its impact on QoL.

Methodology

This observational, descriptive cross-sectional study was conducted from October to December 2024. Adults aged 18 years and above from the Eastern Province were recruited using convenience sampling. A self-administered questionnaire was distributed online. A total of 342 participants were included, after exclusions from the initial 438 responses. Data were analyzed using SPSS.

Results

Of the 342 participants, 239 (69.9%) were female and 103 (30.1%) male. GERD was diagnosed in 79 individuals (23.1%). GERD prevalence was significantly associated with age (p = 0.018), marital status (p = 0.005), and occupation (p = 0.021). No significant association was observed with gender, BMI, or smoking status. GERD symptoms included heartburn in 76 participants (22.2%), food reflux in 55 (16.1%), and sleep disturbance in 53 (15.5%). Participants with GERD had significantly lower QoL scores across all domains. The physical and mental health scores were 3.3 ± 1.2 for GERD participants compared to 3.7 ± 1.1 for those without GERD (p = 0.001). Social relationships scores were 3.3 ± 1.2 versus 3.5 ± 1.2 (p = 0.0001), productivity scores were 3.3 ± 1.1 versus 3.4 ± 1.2 (p = 0.001), and environmental aspect scores were 2.8 ± 1.1 versus 3.0 ± 1.2 (p = 0.017).

Conclusion

GERD is a significant health concern in the Eastern Province, impacting QoL. Although BMI was not significantly associated with GERD in this sample, symptom severity was higher among obese individuals. Increased awareness, early diagnosis, and lifestyle changes, such as weight management, physical activity, and dietary adjustments, are essential to improve QoL and reduce long-term complications such as esophagitis, Barrett’s esophagus, and strictures resulting from GERD.

## Introduction

In recent years, gastroesophageal reflux disease (GERD) has emerged as a significant global health concern. It is a motility disorder caused by the frequent reflux of stomach contents into the esophagus or oral cavity. Common symptoms of GERD include heartburn, regurgitation of gastric contents into the oropharynx, dysphagia, chest pain, water brash, and globus sensation [1].

The pathophysiology of GERD is multifactorial. It involves transient relaxation and dysfunction of the lower esophageal sphincter (LES) resting tone, delayed gastric emptying, impaired peristalsis, insufficient esophageal acid clearance, reduced salivation, impaired mucosal resistance, and increased intra-abdominal pressure in obese individuals [1].

Several risk factors for GERD have been proposed, with the most established being alcohol consumption, family history of GERD, and high BMI [1].

Management of GERD requires a stepwise approach, with the primary goals being symptom control, healing of esophagitis, and prevention of complications or recurrence. Treatment includes lifestyle modification, acid suppression medications (such as antacids or proton pump inhibitors), and in certain cases, surgical intervention through antireflux procedures [2].

Pharmacological treatment is effective for approximately 80% of patients with recurrent but non-progressive GERD. However, early identification of the remaining 20% with progressive disease is critical, as they may develop serious complications such as Barrett’s esophagus or esophageal strictures. Surgical intervention is recommended early in such cases to prevent severe outcomes [2].

Our study was driven by the limited number of studies on GERD in the Eastern Region of Saudi Arabia and the observation that GERD prevalence is higher in Saudi Arabia compared to Western and East Asian countries. While the global prevalence of GERD ranges from 10% to 25%, reported rates in Saudi Arabia range from 20% to 61%, specifically, 20.4% in the Eastern Province, 61.8% in Arar, and 45.4% in Riyadh [3].

Additionally, GERD has been found to be more prevalent in older adults and is associated with several risk factors, including sociodemographic characteristics, smoking, family history, high BMI, types of food and beverages, fast food diets, physical inactivity, and various health conditions. Food and dietary habits have been linked to GERD in numerous studies. Spicy foods, in particular, are known triggers, and one study observed a negative association between coffee consumption and GERD symptoms [4].

High BMI is considered a major risk factor in the development of GERD. Cross-sectional epidemiological studies have consistently shown that obese individuals are more likely to experience GERD than those with a normal BMI [5].

Therefore, the purpose of this study is to increase awareness of the relationship between GERD and obesity. Given the scarcity of published research on this topic in the Eastern Region, and the lack of studies that include a control group, we aimed to address this gap by including both obese and non-obese participants. We also focused on the relationship between meal-to-sleep intervals and GERD risk and severity, an aspect that was overlooked in a previous cross-sectional study conducted in the Eastern Province of Saudi Arabia [6].

## Materials and methods

This study was an observational, descriptive, cross-sectional study conducted across various cities in the Eastern Province of Saudi Arabia, including Dammam, Aseer Province, Al-Khobar, Al-Jubail, Hofuf, and Ras Tanura. Data collection was carried out over a three-month period, from October 2024 to December 2024. The study population included male and female participants aged 18 years and older, both Saudi and non-Saudi individuals, with a BMI of 30 kg/m² or higher. Only residents of the Eastern Province were included, while pregnant women were excluded. The estimated sample size was 314 participants, calculated using Epi Info software version 7.2 (Centers for Disease Control and Prevention, Atlanta, GA, USA), based on a 95% confidence level and a 5% margin of error. A convenience sampling technique was used to recruit participants.

Data collection methods, instruments used, and measurements

A self-administered questionnaire was distributed through Google Forms via social media platforms, including WhatsApp, Telegram, and X (formerly Twitter). To maximize reach and participation from the target population, periodic announcements were posted to reshare the questionnaire link on these platforms. The questionnaire consisted of six sections. Sections one to four collected socio-demographic information, including gender, age, nationality, pregnancy status, place of residence, marital status, educational level, monthly income, occupation, body weight, height, and smoking status. The fifth section assessed whether the participant had been diagnosed with GERD, while the sixth section measured and evaluated GERD symptoms, their duration, and the presence of additional symptoms. The questionnaire was adapted from a previous study on GERD prevalence conducted in Abha [4]. Additionally, seven questions were added to address limitations noted in recent research on GERD in the Eastern Province of Saudi Arabia [6]. BMI was calculated by dividing weight in kilograms by the square of height in meters. The classification of BMI followed WHO guidelines: underweight (<18.5), healthy weight (18.5-24.9), overweight (25-29.9), and obese (≥30) [7].

A pilot study was conducted with ten individuals and staff members to identify any ambiguous or confusing items. Following the pilot, three experts reviewed the questionnaire to assess its validity and reliability. One week later, the questionnaire was re-administered to the same pilot participants to evaluate test-retest reliability.

Data management and analysis plan

The data were entered into a Microsoft Excel sheet and subsequently analyzed using the SPSS. Data from participants involved in the pilot study, as well as those who met the exclusion criteria, were not included in the final analysis. Categorical variables were summarized as frequencies (numbers and percentages), while measures of central tendency (mean, median) were calculated for continuous variables. A p-value of <0.05 was considered statistically significant. Chi-square tests were used to assess whether there were significant differences between the expected and observed frequencies across categories.

Statistical analysis

The data were cleaned, managed, and coded using Microsoft Excel 2019 (Microsoft Corporation, Redmond, WA). Statistical analyses were conducted using R (RStudio, version 1.4.1106; RStudio, Inc.). Descriptive statistics were generated, including frequency distributions and cross-tabulations, which were assessed using the chi-square test. Confidence intervals (95% CI) were calculated, and multivariate logistic regression analysis was applied to identify predictors of the dependent variables. A p-value of less than 0.05 was considered statistically significant.

Ethical considerations

Ethical approval for the study was obtained from the Ethics Committee of King Faisal University (#KFU-REC-2024-SEP-ETHICS2604). Participation in the study was entirely voluntary, with no incentives or rewards offered. All participants were informed about the purpose of the study and their right to refuse participation or withdraw at any point without consequence. Confidentiality and privacy of all participants were maintained throughout the research process.

## Results

Out of 438 participants, 342 were included after exclusions. As presented in Table 1, the majority were female (239; 69.9%) and aged 18-29 years (179; 52.3%). Most participants were Saudi nationals (309; 90.4%) and were classified as having average weight (134; 39.2%), followed by overweight (96; 28.1%) and obese (89; 26.0%). Regarding marital status, 173 (50.6%) were single, while 158 (46.2%) were married. The majority held a university degree (243; 71.1%). Occupationally, 115 (33.6%) were students, followed by participants employed in the government (80; 23.4%) and private sectors (55; 16.1%). Income levels showed that 173 (50.6%) earned less than 5,000 SAR per month. Regarding smoking status, 265 (77.5%) of participants were non-smokers. Finally, 79 (23.1%) of the participants were diagnosed with GERD.

**Table 1:** Sociodemographic characteristics of the study population. GERD: Gastroesophageal reflux disease.

Category	Subcategory	n (%)
Gender	Female	239 (69.9%)
	Male	103 (30.1%)
Age Category	18-29	179 (52.3%)
	30-44	101 (29.5%)
	45-59	53 (15.5%)
	60+	9 (2.6%)
Nationality	Non-Saudi	33 (9.6%)
	Saudi	309 (90.4%)
BMI Category	Underweight (<18.5)	23 (6.7%)
	Average weight (18.5-24.9)	134 (39.2%)
	Overweight (25-29.9)	96 (28.1%)
	Obese (≥30)	89 (26.0%)
Marital Status	Single	173 (50.6%)
	Married	158 (46.2%)
	Divorced	10 (2.9%)
	Widow	1 (0.3%)
Educational Level	Elementary	1 (0.3%)
	Middle school	5 (1.5%)
	High school	68 (19.9%)
	University	243 (71.1%)
	Postgraduate	25 (7.3%)
Occupation	Student	115 (33.6%)
	Government sector	80 (23.4%)
	Private sector	55 (16.1%)
	Unemployed	79 (23.1%)
	Freelancer	13 (3.8%)
Income	Less than 5000	173 (50.6%)
	5000-10000	73 (21.3%)
	More than 10000	96 (28.1%)
Smoking Status	Non-smoker	265 (77.5%)
	Current smoker	27 (7.9%)
	Ex-smoker	25 (7.3%)
	Passive smoker	25 (7.3%)
GERD Diagnosis	Yes	79 (23.1%)
	No	263 (76.9%)

The prevalence of GERD symptoms among the 342 participants is presented in Table 2. Overall, 153 (44.7%) participants reported no epigastric pain, including 141 (41.2%) without GERD and 12 (3.5%) with GERD. Frequent epigastric pain (more than once per week) affected 98 (28.7%) participants, equally distributed between the GERD and non-GERD groups (49; 14.3% each). Food reflux occurring more than twice per week was reported by 55 (16.1%) participants, with a higher prevalence among those with GERD (40; 11.7%) compared to non-GERD participants (15; 4.4%). Heartburn was reported more than twice per week by 76 (22.2%) participants, predominantly among the GERD group (53; 15.5%) compared to the non-GERD group (23; 6.7%).

**Table 2:** The prevalence of GERD symptoms among participants. GERD: Gastroesophageal reflux disease.

Symptom	Frequency	No GERD N (%)	Yes GERD N (%)
Epigastric pain or burning	Every two weeks	23 (6.73%)	16 (4.68%)
	More than once a day	12 (3.51%)	21 (6.14%)
	More than once a week	37 (10.82%)	28 (8.19%)
	Once a month	50 (14.62%)	2 (0.58%)
	No symptoms	141 (41.23%)	12 (3.51%)
Reflux of food into the throat	Every two weeks	25 (7.31%)	10 (2.92%)
	More than once a day	7 (2.05%)	14 (4.09%)
	More than once a week	29 (8.48%)	26 (7.60%)
	Once a month	52 (15.20%)	11 (3.22%)
	No symptoms	150 (43.86%)	18 (5.26%)
Heartburn	Every two weeks	27 (7.89%)	11 (3.22%)
	More than once a day	17 (4.97%)	21 (6.14%)
	More than once a week	44 (12.87%)	32 (9.36%)
	Once a month	50 (14.62%)	3 (0.88%)
	No symptoms	125 (36.55%)	12 (3.51%)
Chest pain	Every two weeks	18 (5.26%)	6 (1.75%)
	More than once a day	10 (2.92%)	10 (2.92%)
	More than once a week	12 (3.51%)	17 (4.97%)
	Once a month	24 (7.02%)	5 (1.46%)
	No symptoms	199 (58.19%)	41 (11.99%)
Chronic cough	Every two weeks	11 (3.22%)	4 (1.17%)
	More than once a day	6 (1.75%)	14 (4.09%)
	More than once a week	10 (2.92%)	12 (3.51%)
	Once a month	16 (4.68%)	4 (1.17%)
	No symptoms	220 (64.33%)	45 (13.16%)
Change in voice	Every two weeks	7 (2.05%)	5 (1.46%)
	More than once a day	5 (1.46%)	10 (2.92%)
	More than once a week	7 (2.05%)	12 (3.51%)
	Once a month	19 (5.56%)	9 (2.63%)
	No symptoms	225 (65.79%)	43 (12.57%)
Sleep disturbance	Every two weeks	22 (6.43%)	8 (2.34%)
	More than once a day	24 (7.02%)	23 (6.73%)
	More than once a week	50 (14.62%)	29 (8.48%)
	Once a month	32 (9.36%)	3 (0.88%)
	No symptoms	135 (39.47%)	16 (4.68%)
Nausea and vomiting	Every two weeks	18 (5.26%)	9 (2.63%)
	More than once a day	10 (2.92%)	16 (4.68%)
	More than once a week	28 (8.19%)	11 (3.22%)
	Once a month	28 (8.19%)	10 (2.92%)
	No symptoms	179 (52.34%)	33 (9.65%)
Dysphagia	Every two weeks	14 (77.78%)	4 (22.22%)
	More than once a day	6 (28.57%)	15 (71.43%)
	More than once a week	12 (52.17%)	11 (47.83%)
	Once a month	12 (66.67%)	6 (33.33%)
	No symptoms	219 (83.59%)	43 (16.41%)
Sore throat	Every two weeks	9 (47.37%)	10 (52.63%)
	More than once a day	7 (38.89%)	11 (61.11%)
	More than once a week	12 (60.00%)	8 (40.00%)
	Once a month	31 (70.45%)	13 (29.55%)
	No symptoms	204 (84.65%)	37 (15.35%)
Dyspepsia	Every two weeks	13 (54.17%)	11 (45.83%)
	More than once a day	22 (51.16%)	21 (48.84%)
	More than once a week	26 (50.98%)	25 (49.02%)
	Once a month	19 (95.00%)	1 (5.00%)
	No symptoms	183 (89.71%)	21 (10.29%)
Weakness or fatigue	Every two weeks	18 (72.00%)	7 (28.00%)
	More than once a day	31 (47.69%)	34 (52.31%)
	More than once a week	39 (72.22%)	15 (27.78%)
	Once a month	23 (92.00%)	2 (8.00%)
	No symptoms	152 (87.86%)	21 (12.14%)
Loss of appetite	Every two weeks	17 (70.83%)	7 (29.17%)
	More than once a day	14 (41.18%)	20 (58.82%)
	More than once a week	26 (57.78%)	19 (42.22%)
	Once a month	28 (87.50%)	4 (12.50%)
	No symptoms	178 (86.23%)	29 (13.77%)
Bloating	Every two weeks	17 (73.91%)	6 (26.09%)
	More than once a day	31 (48.44%)	33 (51.56%)
	More than once a week	38 (67.86%)	18 (32.14%)
	Once a month	25 (92.59%)	2 (7.41%)
	No symptoms	152 (88.37%)	20 (11.63%)
Diarrhea	Every two weeks	17 (68.00%)	8 (32.00%)
	More than once a day	12 (48.00%)	13 (52.00%)
	More than once a week	14 (56.00%)	11 (44.00%)
	Once a month	27 (84.38%)	5 (15.63%)
	No symptoms	193 (82.13%)	42 (17.87%)
Constipation	Every two weeks	22 (75.86%)	7 (24.14%)
	More than once a day	21 (56.76%)	16 (43.24%)
	More than once a week	29 (61.70%)	18 (38.30%)
	Once a month	29 (85.29%)	5 (14.71%)
	No symptoms	162 (83.08%)	33 (16.92%)

Table 3 shows that symptom duration varied among participants. Of the 342 participants, 64 (18.7%) had no GERD symptoms. Short-term symptoms (<6 months) were reported by 45 (13.2%), while 103 (30.1%) had symptoms lasting 6-12 months. A duration of 1-3 years was reported by 83 (24.3%), and 65 (19.0%) experienced symptoms for more than three years. Notably, long-term symptoms (>3 years) were nearly equally distributed between the GERD (33; 50.8%) and non-GERD (32; 49.2%) groups.

**Table 3:** Overall duration of GERD symptoms among the population. GERD: Gastroesophageal reflux disease.

Duration of Symptoms	No GERD (n, %)	Yes GERD (n, %)	Total
Less than six months	31 (68.89%)	14 (31.11%)	45
Six months to 1 year	99 (96.12%)	4 (3.88%)	103
One year to 3 years	68 (81.93%)	15 (18.07%)	83
More than three years	32 (49.23%)	33 (50.77%)	65
I had no symptoms	33 (71.74%)	13 (28.26%)	46
Total	263	79	342

Table 4 shows that GERD was significantly associated with age (p = 0.018, Cramér’s V = 0.17), marital status (p = 0.005, Cramér’s V = 0.19), and occupation (p = 0.021, Cramér’s V = 0.18). Higher GERD rates were observed in the 30-44 age group, among married individuals, and those who were unemployed or employed in the government sector. Other factors such as gender, nationality, BMI, education, income, and smoking status showed no significant association with GERD (p > 0.05), though slightly higher GERD prevalence was noted among non-smokers and those with lower income.

**Table 4:** Association between GERD and sociodemographic factors. GERD: Gastroesophageal reflux disease.

Variable Category	No GERD (N (%)	Yes GERD N (%)	Total N (%)	p-value
Gender				0.616
Female	182 (53.2%)	57 (16.7%)	239 (69.9%)	
Male	81 (23.7%)	22 (6.4%)	103 (30.1%)	
Total	263 (76.9%)	79 (23.1%)	342 (100%)	
Age Category				
18-29	150 (43.9%)	29 (8.5%)	179 (52.3%)	0.018
30-44	70 (20.5%)	31 (9.1%)	101 (29.5%)	
45-59	37 (10.8%)	16 (4.7%)	53 (15.5%)	
60+	6 (1.8%)	3 (0.9%)	9 (2.6%)	
Total	263 (76.9%)	79 (23.1%)	342 (100%)	
Nationality				
Non-Saudi	25 (7.3%)	8 (2.3%)	33 (9.6%)	
Saudi	238 (69.6%)	71 (20.8%)	309 (90.4%)	
Total	263 (76.9%)	79 (23.1%)	342 (100%)	0.87
BMI Category				
Normal weight (18.5-24.9)	103 (30.1%)	31 (9.1%)	134 (39.2%)	0.638
Obese (>=30)	71 (20.8%)	18 (5.3%)	89 (26.0%)	
Overweight (25-29.9)	70 (20.5%)	26 (7.6%)	96 (28.1%)	
Underweight (<18.5)	19 (5.6%)	4 (1.2%)	23 (6.7%)	
Total	263 (76.9%)	79 (23.1%)	342 (100%)	
Marital Status				
Divorced	6 (1.8%)	4 (1.2%)	10 (2.9%)	0.005
Married	112 (32.7%)	46 (13.5%)	158 (46.2%)	
Single	145 (42.4%)	28 (8.2%)	173 (50.6%)	
Widow	0 (0.0%)	1 (0.3%)	1 (0.3%)	
Total	263 (76.9%)	79 (23.1%)	342 (100%)	
Educational Level				
Elementary	0 (0.0%)	1 (0.3%)	1 (0.3%)	0.263
High school	51 (14.9%)	17 (5.0%)	68 (19.9%)	
Middle school	4 (1.2%)	1 (0.3%)	5 (1.5%)	
Postgraduate	22 (6.4%)	3 (0.9%)	25 (7.3%)	
University	186 (54.4%)	57 (16.7%)	243 (71.1%)	
Total	263 (76.9%)	79 (23.1%)	342 (100%)	
Occupation				
Freelancer	10 (2.9%)	3 (0.9%)	13 (3.8%)	0.021
Government sector	60 (17.5%)	20 (5.8%)	80 (23.4%)	
Private sector	40 (11.7%)	15 (4.4%)	55 (16.1%)	
Student	100 (29.2%)	15 (4.4%)	115 (33.6%)	
Unemployed	53 (15.5%)	26 (7.6%)	79 (23.1%)	
Total	263 (76.9%)	79 (23.1%)	342 (100%)	
Income				
5000-10000	49 (14.3%)	24 (7.0%)	73 (21.3%)	0.064
Less than 5000	140 (40.9%)	33 (9.6%)	173 (50.6%)	
More than 10000	74 (21.6%)	22 (6.4%)	96 (28.1%)	
Total	263 (76.9%)	79 (23.1%)	342 (100%)	
Smoking Status				
Current smoker	19 (5.6%)	8 (2.3%)	27 (7.9%)	0.542
Ex-smoker	17 (5.0%)	8 (2.3%)	25 (7.3%)	
Non-smoker	208 (60.8%)	57 (16.7%)	265 (77.5%)	
Passive smoker	19 (5.6%)	6 (1.8%)	25 (7.3%)	
Total	263 (76.9%)	79 (23.1%)	342 (100%)	

Comparisons of different quality of life (QoL) domains and sociodemographic characteristics are shown in Table 5. Participants with GERD had significantly lower scores in physical and mental health (3.3 ± 1.2 vs. 3.7 ± 1.1, p = 0.001), social life and relationships (3.3 ± 1.2 vs. 3.5 ± 1.2, p = 0.0001), and personal abilities and productivity (3.3 ± 1.1 vs. 3.4 ± 1.2, p = 0.001). Environmental aspect scores were also lower among the GERD group (2.8 ± 1.1 vs. 3.0 ± 1.2, p = 0.017).

**Table 5:** Comparison of quality of life (QOL) domains by sociodemographic characteristics in the study population. GERD: Gastroesophageal reflux disease.

Domains of QOL	GERD Mean ± SD	Non-GERD Mean ± SD	p-value
Physical and Mental Health	3.3 ± 1.2	3.7 ± 1.1	0.001
Social Life and Relationships	3.3 ± 1.2	3.5 ± 1.2	0.0001
Personal Abilities and Productivity	3.3 ± 1.1	3.4 ± 1.2	0.001
Environmental Aspects	2.8 ± 1.1	3.0 ± 1.2	0.017

**Figure 1:**
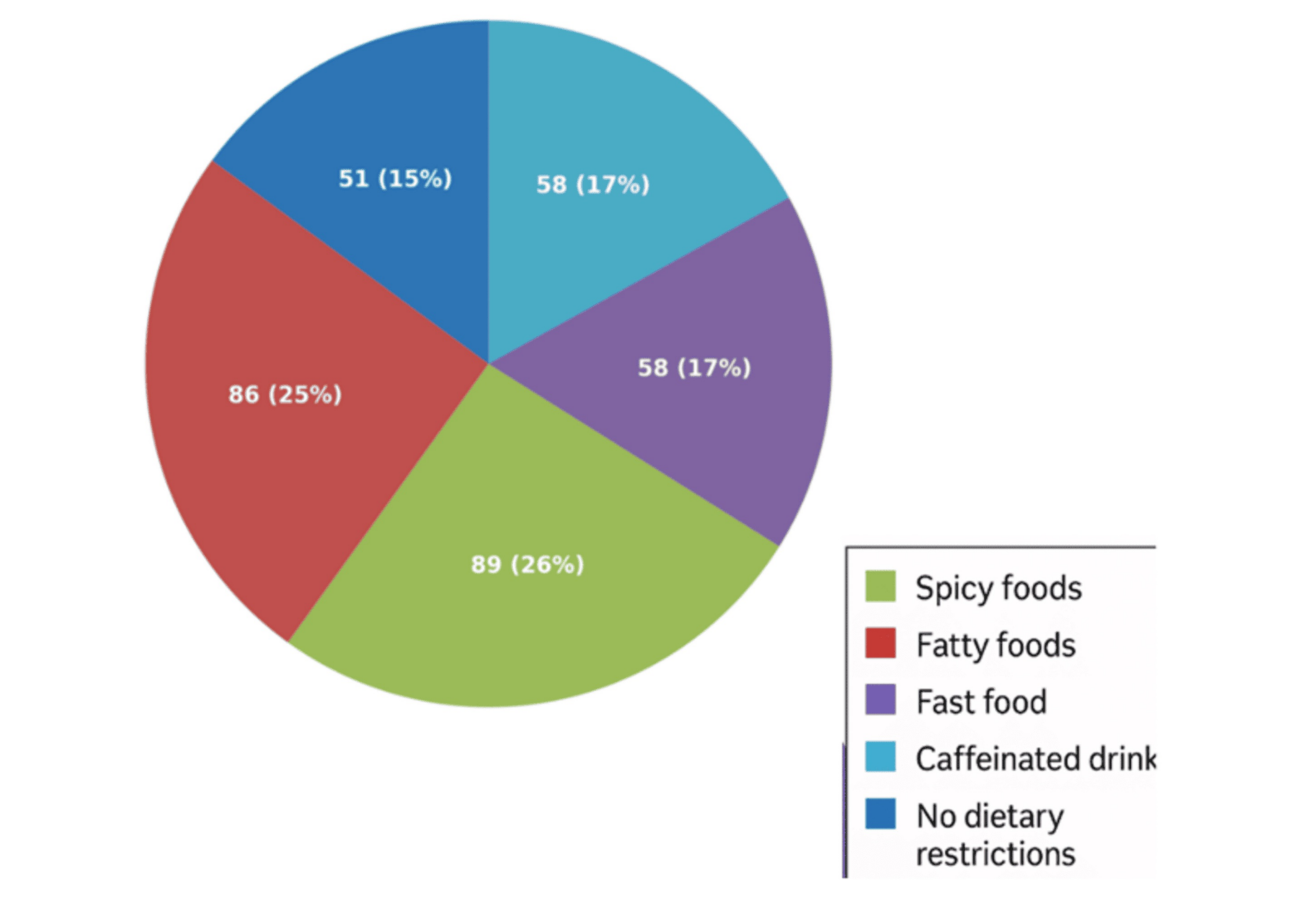
Patterns of food avoidance among study participants.

As illustrated in Figure 1, food avoidance patterns among study participants (N = 342) revealed that spicy foods were the most frequently avoided dietary category (89; 26%), followed closely by fatty foods (86; 25.1%). Equal proportions of participants reported avoiding fast food and caffeinated beverages/soft drinks (58; 17%) each. A smaller subset (51; 14.9%) reported no dietary restrictions from the listed categories.

**Figure 2:**
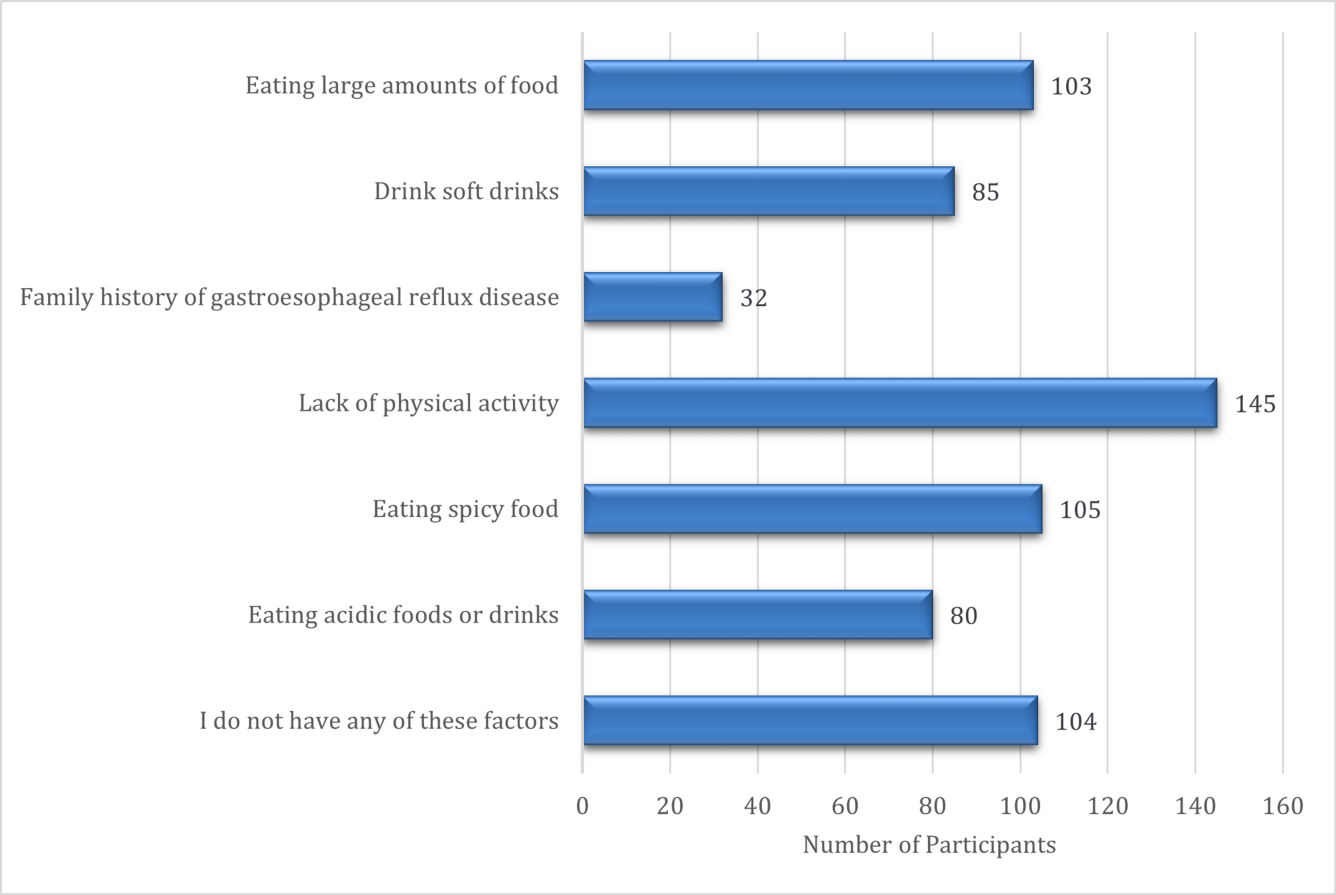
Risk factors of GERD among the participants. GERD: Gastroesophageal reflux disease.

Figure 2 presents the self-reported risk factors associated with GERD among study participants. The most frequently identified risk factor was lack of physical activity (n = 145), followed by eating spicy food (n = 105), eating large amounts of food (n = 103), and the absence of any identifiable risk factors (n = 104). Other commonly reported factors included drinking soft drinks (n = 85) and consuming acidic foods or beverages (n = 80). Only 32 participants reported a family history of GERD as a risk factor. These findings emphasize the importance of lifestyle-related behaviors, particularly diet and physical activity, in the development and management of GERD.

## Discussion

This study investigated the prevalence, risk factors, and effects of GERD among 342 mostly young Saudi nationals, with a significant majority being female (69.9%) and well-educated (71.1% possessing university degrees). The results underscore a substantial burden of GERD symptoms, correlations with lifestyle and sociodemographic characteristics, and a marked deterioration in QOL among those affected.

The research indicated a significant prevalence of GERD symptoms, with 22.2% of subjects reporting recurrent heartburn and 16.1% experiencing food reflux. These findings correspond with earlier research in Saudi Arabia, indicating a GERD prevalence between 20% and 45%, highlighting an escalating health issue in the region [8]. Chronic cough, dysphagia, and sleep problems were prevalent among GERD patients, aligning with global literature that associates GERD with extra-esophageal symptoms [9]. A significant percentage of individuals (26%) abstained from spicy meals and 25% from fatty foods, indicating that dietary alterations are a common self-management tactic; however, therapeutic advice may be required for optimal symptom regulation.

Identified key risk variables included physical inactivity, reported by 145 participants, and the consumption of spicy foods. These findings align with global studies linking sedentary lifestyles and specific dietary practices to a heightened risk of GERD [10]. The study found that 28.1% of participants were overweight and 26% were obese, supporting the link between obesity and GERD, which is attributed to increased intra-abdominal pressure and dysfunction of the lower esophageal sphincter [11]. However, this study did not identify a statistically significant correlation between BMI and GERD, in contrast to some previous research, potentially due to the younger age demographic or sample characteristics.

Age and marital status showed a significant correlation with GERD (p = 0.018 and p = 0.005, respectively), with older and married individuals demonstrating a higher prevalence of symptoms. This may be attributed to lifestyle modifications, stress, or dietary habits associated with married life [12]. Notably, gender, educational attainment, and wealth did not exhibit significant correlations, contrary to several studies indicating a higher frequency of GERD among males or individuals from lower socioeconomic strata [13]. This discrepancy may be due to the predominantly young, female, and educated sample in this study.

A troubling discovery was that 50.8% of GERD participants reported symptoms persisting for over three years, suggesting chronicity and possible inadequate treatment. In contrast, 71.7% of individuals without GERD reported no symptoms, highlighting the importance of early identification and management of GERD to avoid complications such as esophagitis or Barrett’s esophagus [14]. The significant prevalence of chronic sufferers may indicate deficiencies in healthcare access, awareness, or treatment compliance.

GERD markedly reduced participants’ QoL, especially in the physical and mental health domains (p = 0.001). The adverse impacts on social life, productivity, and general well-being correspond with global evidence indicating that GERD diminishes work performance and daily functioning [15]. The research emphasizes the necessity for comprehensive GERD management, incorporating nutritional guidance, lifestyle changes, and psychological support to alleviate these effects.

Limitations

This study has several limitations, notably its cross-sectional design, which prevents causal inferences. The predominance of young, educated women may limit generalizability to other demographic groups. Moreover, the use of self-reported symptoms introduces the possibility of recall bias. Future long-term research using diverse samples and objective diagnostic methods (e.g., endoscopy) could enhance the validity of these findings.

## Conclusions

GERD is a common and burdensome condition among young Saudi individuals, significantly affecting their quality of life. While key modifiable risk factors include dietary habits and physical inactivity, this study also identified a significant association between GERD and both age and marital status. Public health strategies that encourage healthier lifestyles, early medical evaluation, and comprehensive GERD management are essential to alleviating its burden. Future research should focus on long-term outcomes and effective treatment strategies in similar populations.
